# Therapy with high-dose Interleukin-2 (HD IL-2) in metastatic melanoma and renal cell carcinoma following PD1 or PDL1 inhibition

**DOI:** 10.1186/s40425-019-0522-3

**Published:** 2019-02-18

**Authors:** Elizabeth I. Buchbinder, Janice P. Dutcher, Gregory A. Daniels, Brendan D. Curti, Sapna P. Patel, Shernan G. Holtan, Gerald P. Miletello, Mayer N. Fishman, Rene Gonzalez, Joseph I. Clark, John M. Richart, Christopher D. Lao, Scott S. Tykodi, Ann W. Silk, David F. McDermott

**Affiliations:** 1 Dana Farber Cancer Institute, Boston, MA USA; 2 Cancer Research Foundation of NY, Chappaqua,, NY USA; 3 Moores UCSD Cancer Center, La Jolla, San Diego, CA USA; 4 Providence Health & Services, Portland, OR USA; 5 The University of Texas MD Anderson Cancer Center, Houston, TX 77030 USA; 6 University of Minnesota, Minneapolis, MN USA; 7 Hematology/Oncology Clinic, Baton Rouge, LA USA; 8 Moffitt Cancer Center, Tampa, FL USA; 9 University of Colorado, Aurora, CO USA; 10 Loyola University Stritch School of Medicine, Maywood, IL USA; 11 Saint Louis University, Saint Louis, MO 63110 USA; 12 University of Michigan, Ann Arbor, MI USA; 13 University of Washington and Fred Hutchinson Cancer Research Center, Seattle, WA USA; 14 Rutgers Cancer Institute of New Jersey, New Brunswick, NJ USA; 15 Beth Israel Deaconess Medical Center, Boston, MA USA

## Abstract

**Background:**

Metastatic melanoma (mM) and renal cell carcinoma (mRCC) are often treated with anti-PD-1 based therapy, however not all patients respond and further therapies are needed. High dose interleukin-2 (HD IL-2) can lead to durable responses in a subset of mM and mRCC patients. The efficacy and toxicity of HD IL-2 therapy following anti-PD-1 or anti-PD-L1 therapy have not yet been explored.

**Methods:**

Reports on mM and mRCC patients who had received HD IL-2 after PD-1 or PD-L1 inhibition were queried from the PROCLAIM^SM^ database. Patient characteristics, toxicity and efficacy were analyzed.

**Results:**

A total of 57 patients (40 mM, 17 mRCC) were treated with high dose IL-2 after PD-1 or PD-L1 inhibition and had data recorded in the PROCLAIM database. The best overall response rate to HD IL-2 was 22.5% for mM (4 complete response (CR), 5 partial responses (PRs)) and 24% for mRCC (2 CRs, 2 PRs). The toxicity related to HD IL-2 observed in these patients was similar to that observed in patients treated with HD IL-2 without prior checkpoint blockade. One patient who had received prior PD-L1 blockade developed drug induced pneumonitis with HD IL-2 requiring steroid therapy.

**Conclusion:**

In this retrospective analysis, HD IL-2 therapy displayed durable antitumor activity in mM and mRCC patients who progressed following treatment with PD-1 and PD-L1 inhibition. The toxicities were generally manageable and consistent with expectations from HD IL-2 but physicians should watch for immune related toxicities such as pneumonitis. This analysis supports the development of randomized prospective trials to assess the proper sequencing and combination of immune checkpoint blockade and cytokine therapy.

**Electronic supplementary material:**

The online version of this article (10.1186/s40425-019-0522-3) contains supplementary material, which is available to authorized users.

## Background

Immunotherapy is rapidly expanding into the treatment of numerous malignancies. One of the earliest immunotherapies, high dose interleukin-2 (HD IL-2), activates T-cells and has documented durable tumor responses in a subset of patients with mM and mRCC. [1–3] However, the acute toxicity profile and requirement for intensive inpatient management have limited the application of HD IL-2, and immune checkpoint blockade (ICB) has largely replaced it as a frontline treatment of advanced mM and mRCC. [4]

Ipilimumab, has proven benefit in metastatic melanoma (mM) as a single agent and now in combination with other immunotherapy agents. PD-1 inhibition with nivolumab or pembrolizumab has been even more effective leading to FDA approval in mM, renal cell carcinoma (mRCC), and several other malignancies. [5–10] However, alternative therapies are needed for patients who develop severe side effects, progress after an initial response or fail to respond to ICB.

The field is plentiful with clinical trials of novel agents targeting immune checkpoints, injectable therapies such as anti-tumor viruses, T-cell based therapies including TIL (Tumor infiltrating lymphocyte) and CAR-T cells (Chimeric antigen receptor T-cells) alone and in combination. With this explosion of interest in immunotherapy, there has been renewed interest in cytokines and their role in immune stimulation and overcoming resistance to checkpoint inhibition. Novel drugs targeting the IL-2 receptor are in clinical trials.

The field of immunotherapy is now tasked with finding appropriate treatments for patients who do not benefit from ICB. Clinical trials involving single agents and combinations require hypothesis-generating data as well as clinical experience to help guide progress in this area.

In this study, we queried the PROCLAIM database for patients with mM or mRCC who had developed resistance to PD-1/PD-L1 inhibition and were subsequently treated with HD IL-2 to examine its efficacy, toxicity and long-term outcomes in the salvage setting. We also queried the database for patients treated with HD IL-2 who did not receive prior PD-1 or PD-L1 inhibition as a comparator group.

## Methods

### Patients

The PROCLAIM registry is a database of patients, from more than 40 community and large academic centers, who received HD IL-2 in the treatment of mM or mRCC (Clinicaltrials.gov: NCT 01415167) The study was approved by the institutional review boards of the sites enrolling subjects and all patients provided written, informed consent. For this study, the registry was queried to identify patients treated with HD IL-2 prior to or following PD-1 or PD-L1 inhibition. Patient characteristics, including age, gender, disease type, number and type of prior therapies and prior responses to therapy by treating physician assessment were analyzed. In addition, the IL-2 dosing, response and toxicity were reported.

### Treatment

HD IL-2 was administered per the treating institution’s standard of care as an inpatient regimen, typically utilizing a 600,000 IU/kg or 720,000 IU/kg IV infusion every 8 h as tolerated up to 14 consecutive doses over 5 days. The database captured up to 3 toxicities leading to discontinuation of IL-2 per patient. Those patients who were admitted for a second week/cycle of treatment, returned after approximately 9 days off therapy. Two weeks of HD IL-2 therapy constituted one course of treatment. Some patients received additional courses of therapy per the treating physician’s discretion.

### Response data and toxicity

The number of patients who achieved a complete response (CR), partial responses (PR), stable disease (SD) and progressive disease (PD) at initial assessment following HD IL-2, as determined by the treating physician using RECIST 1.1 is reported. In addition, best overall response, objective response rate (ORR) and median overall survival (OS) are also reported. The best overall response is the best response recorded from the start of HD IL-2 treatment until disease progression/recurrence or initiation of a new anti-cancer therapy. Response and survival endpoints were measured from the start of HD IL-2 therapy and were compared amongst patients who did and those who did not receive prior PD-1 or PD-L1 therapy. Toxicities, and immune related toxicities, were examined in patients who received IL-2 after PD-1 or PD-L1 inhibition and compared to subjects who received HD IL-2 without prior ICB.

### Statistical analysis

All statistical analyses were performed using SAS software version 9.4 (SAS Institute, Cary, NC). Frequency counts and measures of central tendency were performed to provide descriptive statistics; medians were reported with the minimum and maximum values. Kaplan-Meier curves with 95% confidence intervals (CIs) were used to estimate median overall survival (mOS) and progression free survival (PFS). Overall survival time was calculated from the date of first dose of HD IL-2 to either the date of death or date of most recent follow-up. PFS was calculated from the date of first dose of HD IL-2 to the date of mM or mRCC progression or start of a new anti-cancer treatment.

## Results

### Patient characteristics

A total of 57 patients (40 mM, 17 mRCC) were identified within the PROCLAIM registry who had received high dose IL-2 following treatment with PD-1 or PD-L1 inhibition. Another 1122 mM and mRCC patients were identified within the registry who were treated with HD IL-2 without prior ICB.

Among the mM patients, 18 received PD-1 inhibition with nivolumab, 17 with pembrolizumab, and the remainder received PD-1 inhibition without identification of the drug. Four mM patients received combination nivolumab and ipilimumab. Among the mRCC patients 8 received PD-1 inhibition with nivolumab and 1 received pembrolizumab. Three mRCC patients received PD-L1 inhibition with atezolizumab and the remainder received PD-1 inhibition without identification of the drug. Patients may have received more than one PD-1 or PD-L1 targeted agent. Patients were predominantly male with ECOG performance status of 0 or 1 and had greater than or equal to 3 sites of metastatic disease (Additional file 1: Table S1). The mean LDH was 336.5 (117–904) in the mM patients.

Mutational status was reported for 25% of all 40 mM patients. Among those patients in whom mutational status was reported, 4 were noted to be BRAF mutant, 5 were noted to be NRAS mutant and one had a cKIT mutation. Among the mM patients, seven had prior BRAF inhibitor therapy. Among the mRCC patients, six had prior anti-VEGF tyrosine kinase inhibitor therapy.

The mean time between diagnosis of metastatic disease and initiation of HD IL-2 was 22.4 (0.8–55.8) months for the mM patients and 31.8 (0.2–156.0) months for the mRCC patients. The average treatment duration for HD IL-2 (including rest periods) was 2.1 (0.1–7.3) months in the mM patients and 1.8 (0.1–6.1) in the mRCC patients. All patients had progression of their disease prior to initiating treatment with HD IL-2.

The average time between completing PD-1/PD-L1 therapy and starting HD IL-2 was 6.3 (0.3–28.1) months for the mM patients, and 2.1 (0.4–7.4) months for the mRCC patients. On average mM patients were on PD-1/PD-L1 therapy for a mean of 5.4 (1.5–27.5) months and 9.3 (0.7–31.2) months in the mRCC patients. In mM patients, investigator reported response data to PD-1/PD-L1 therapy was available for 29 patients, all of whom had PD. Among the mRCC patients, investigator reported response data to PD-1/PD-L1 therapy was available for 12 patients, one patient had an initial PR but eventually progressed and 11 patients had PD.

### Toxicity

Adverse events reported prior to initiation of HD IL-2 include one mM patient and one mRCC with elevated liver function testing prior to initiation of HD IL-2. Prior toxicity to PD-1 and PD-L1 was recorded retrospectively so some of the data may be lacking.

Hypotension was the most common toxicity reported which resulted in the cessation of HD IL-2. Other recorded toxicities include tachycardia, diarrhea, hypoxia, thrombocytopenia, rigors, capillary leak syndrome, confusion, mental fatigue and pruritus. These toxicities are consistent with those observed with HD IL-2. (Additional file 2: Table S2).

The average number of HD IL-2 doses per cycle is often used as a reflection of toxicity since IL-2 is held when a patient is having more severe side effects. A course of high dose IL-2 therapy consists of two cycles. The mM patients received an average of 8.1 (SD 2.4) doses of IL-2 per cycle. The mRCC patients received an average of 8.0 (SD 3.0) doses of IL-2 per cycle. Among the mM patients for whom cycle data was available, 20% received one cycle, 27.5% received two cycles, 7.5% received 3 cycles, 22.5% received 4 cycles and 12.5% received more than 4 cycles. In the mRCC patient cohort for whom cycle data was available 12% received one cycle, 35% received 2 cycles, 29% received four cycles and 12% received more than 4 cycles. (Additional file 3: Table S3).

The database reported no autoimmune toxicities in any of the patients receiving HD IL-2 following PD-1 or PD-L1 inhibition. However, one of the institutions reported a patient with presumed, severe pneumonitis during HD IL-2 therapy that was felt to be related to prior PD-L1 inhibition. This patient had no prior irAEs to PD-L1 inhibition. On treatment day 7, after receiving 11 of a possible 14 HD IL-2 doses, he developed shortness of breath and hypoxia with an oxygen saturation of 88%. Initial imaging was consistent with pulmonary edema from HD IL-2 induced capillary leak, however his condition did not improve with diuresis and repeat imaging showed worsening pulmonary infiltrates suggestive of pneumonitis. He was treated with intravenous methylprednisolone to treat presumed immune related pneumonitis and had marked improvement in symptoms, oxygen saturation and imaging. He subsequently underwent a steroid taper with continued improvement in symptoms.

### Response rates and survival

The median follow up of all patients following HD IL-2 treatment was 11.2 (0.3–30.9) months for the mM group and 11.3 (0.6–29.0) months for the mRCC group. Of the patients previously treated with PD-1/PD-L1 inhibition, the best overall response reported to HD IL-2 was 23% (23% in mM and 24% in mRCC) with 6 patients (4 mM and 2 mRCC) experiencing a complete response (CR) and 7 patients (5 mM and 2 mRCC) experiencing a partial response (PR). 38% of the mM patients and 47% of the mRCC patients had stable disease. Response data is summarized in Table 1. None of the melanoma patients who had a CR had progressed at the time of the database lock, 1–4 years of follow up. 3/5 PRs in melanoma were continuing as of the database lock, 1–2 years of follow up. None of the two mRCC patients who had a CR progressed, over two years of follow up. Neither of the two mRCC patients with a PR progressed, over two years of follow up.

**Table 1:** Best Response to HD IL-2

Type	Best Response	mM (*N* = 40)	mRCC (*N* = 17)
Initial Response	Complete Response	0 (0)	1 (6)
	Partial Response	3 (8)	1 (6)
	Stable Disease	20 (50)	9 (53)
	Progressive Disease	17 (42)	6 (35)
Best Overall Response	Complete Response	4 (10)	2 (12)
	Partial Response	5 (13)	2 (12)
	Stable Disease	15 (37)	8 (47)
	Progressive Disease	16 (40)	5 (29)

**Fig. 1:**
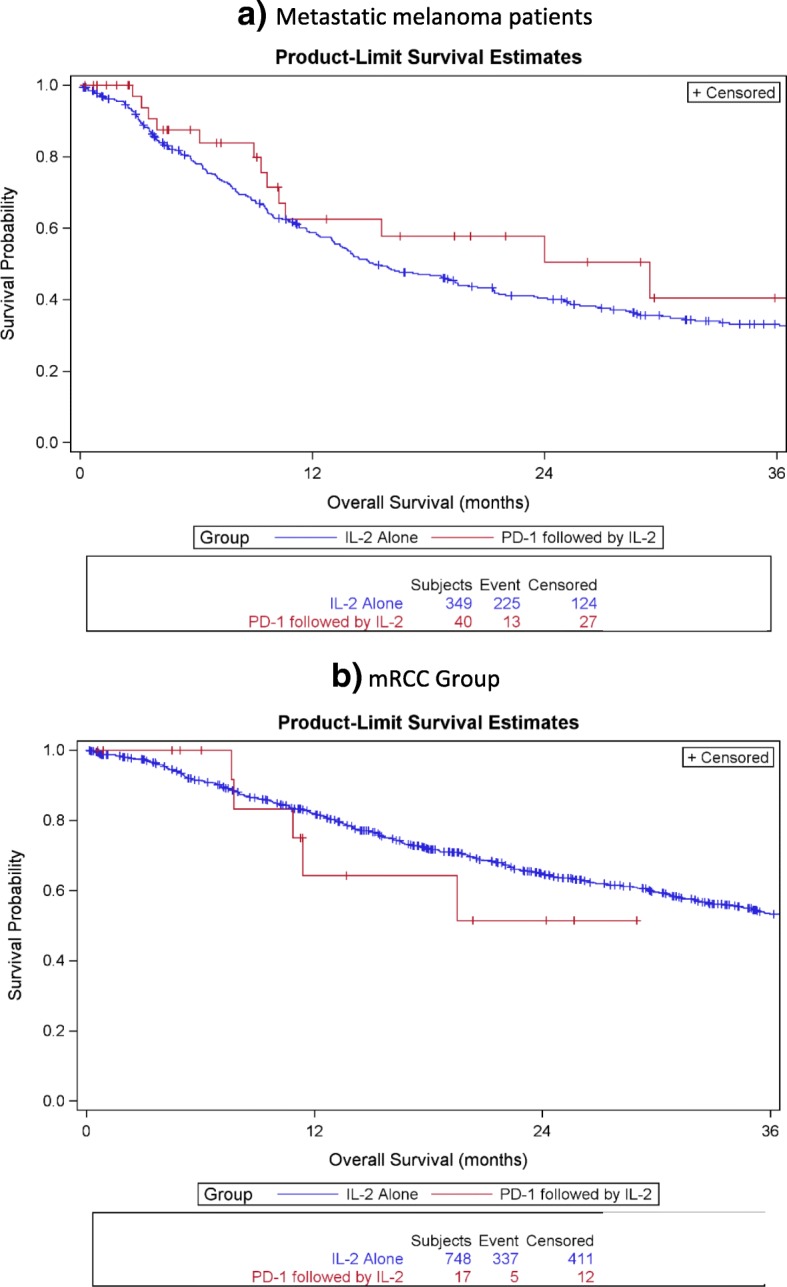
Overall Survival in months for patients treated with IL-2 alone or IL-2 following PD-1 inhibition in metastatic melanoma (**a**) and metastatic renal cell carcinoma (**b**)

**Fig. 2:**
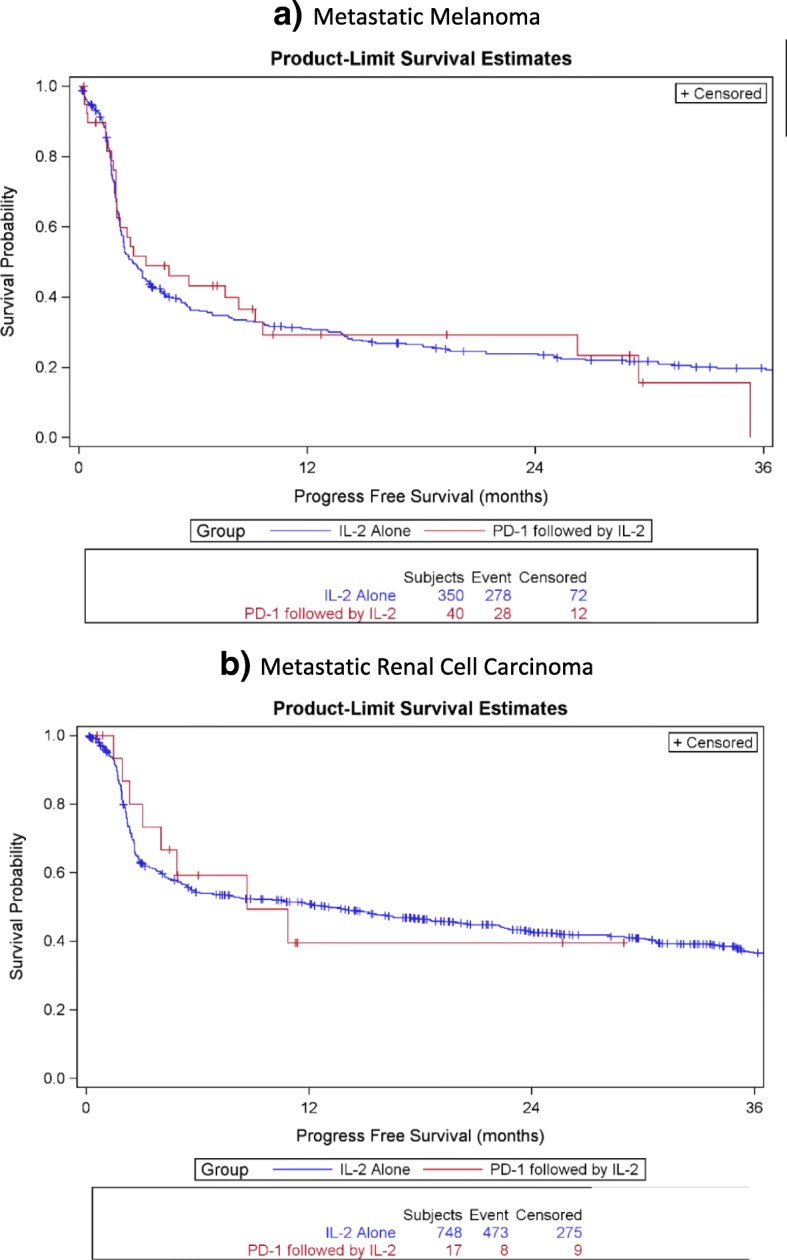
Progression-Free Survival in months for patients treated with IL-2 alone or IL-2 following PD-1 inhibition in metastatic melanoma (**a**) and metastatic renal cell carcinoma (**b**)

The median overall survival for mM patients with prior ICB, measured from the initiation of HD IL-2 administration, was 29.4 months as compared to 15.3 months in the HD IL-2 alone group. The median overall survival for mRCC patients with prior ICB was not reached as compared to 40.8 months in the HD IL-2 alone group. The median progression free survival in the mM group was 3.5 months as compared to 2.8 months in the HD-IL2 alone group and was 8.6 months in the mRCC group as compared to 13 months in the HD IL-2 alone group. Representative curves comparing the two groups are presented in Figs. 1 and 2.

## Discussion

In this analysis, we examined the response and toxicity patterns of patients previously treated with PD-1 or PD-L1 inhibition who then received HD IL-2. We observed a response rate to HD IL-2 consistent with that observed in patients who had not been previously treated with ICB. Although the study cohort in this report is small, the data suggests that HD IL-2 is a viable option for patients who have progressed after ICB. In fact, the overall survival data was very similar for patients who previously received PD-1 or PD-L1 inhibition as those who did not. Since these patients were more heavily pre-treated it is encouraging that these outcomes were similar although there is concern that we are selecting for patients with more slowly progressive disease able to receive multiple treatments.

The success of PD-1 and PD-L1 inhibition in oncology is expanding opportunities to explore combination immunotherapy. HD IL-2 is an attractive target for such exploration since it is a therapy that has documented, single agent anti-tumor efficacy and prolonged duration of responses and survival. [3] In addition, many oncologists already have extensive experience with high dose IL-2 allowing for it to be given safely in the appropriate setting. When studied in a mouse model of chronic lymphocytic choriomeningitis virus it was observed that PD-L1 blockade synergizes with IL-2 therapy to enhance CD8+ T cell responses and decrease viral load. [11] In addition a report of 36 patients who received ipilimumab with IL-2 demonstrated a 17% complete response rate and manageable toxicity. [12]

Experience has demonstrated that combination immunotherapies can have marked increased toxicity as observed with combination ipilimumab and nivolumab [8]. In addition, an increase in toxicity was observed when ipilimumab and nivolumab therapies were sequenced in mM patients suggesting residual effects from prior immunotherapy. [13] [14] In this analysis, there was no obvious trend towards increased toxicity in the group of patients who received HD IL-2 after PD-1 or PD-L1 inhibition. However, there was a case report of a patient with presumed IL-2 induced pneumonitis that required steroid therapy. Pneumonitis during HD IL-2 has not been previously reported. HD IL-2 leads to increased cytokine production leading to T-cell stimulation. In a case of indolent pneumonitis where there are already immune infiltrates within the lung, it is possible that there may be a recall exacerbation when anti-PD1 therapy is followed by HD IL-2. Due to the retrospective nature of this database an analysis such as this might miss other important immune related toxicities that were less severe but clinically important.

One of the potential factors that may be limiting HD IL-2 efficacy is its often-competing actions maintaining T regulatory cells in addition to CD8+ T cells an NK cells that target tumors. Attempts to overcome this have led to the development of novel compounds targeting subunits of the IL-2 receptor. The IL-2 receptor consists of 3 components an α chain, β chain and γ chain with stimulation through βγ leading to stimulation of an immune response and stimulation through αβγ leading to immune suppression [15]. A novel compound which targets the IL-2Rβγ is NKTR-214 which is being tested alone and in combination with ICB (NCT02869295, NCT02983045, NCT03138889). ALKS 4230 is a fusion protein of circularly permuted IL-2 and a soluble portion of IL-2Rα to prevent signaling through IL-2Rα which is also in clinical testing (NCT02799095). Also in clinical testing are FAP-IL-2v and CEA-IL2v, IL-2 variant based immunocytokines (NCT02350673).

These data support continued exploration of HD IL-2 as an immunotherapy in patients with mM and mRCC. There does not appear to be a detrimental outcome for the sequential use of HD IL-2 following anti-PD-1 therapy, and ORR is at least comparable to studies of IL-2 alone. There are currently several studies combining ICB with HD IL-2 including two in mRCC (NCT02989714, NCT0296078) and one in mM (NCT02748564). In addition, studies of novel compounds targeting IL-2 are an important addition to our current exploration of immunotherapy and will build on our understanding of immune signaling.

## Additional files

Additional file 1: **Tables S1.** Patient Demographics (DOCX 31 kb)

Additional file 2: **Tables S2.** Adverse Events Limiting IL-2 Administration (By Frequency) – supplemental materials (DOCX 32 kb)

Additional file 3: **Tables S3.** Duration of IL-2 Therapy (DOCX 20 kb)

## References

[1] Atkins, M.B., et al., High-dose recombinant interleukin 2 therapy for patients with metastatic melanoma: analysis of 270 patients treated between 1985 and 1993*.* J Clin Oncol, 1999. 17(7): p. 2105–2116. 10.1200/JCO.1999.17.7.2105 10561265

[2] Fyfe G (1995). Results of treatment of 255 patients with metastatic renal cell carcinoma who received high-dose recombinant interleukin-2 therapy. J Clin Oncol.

[3] Clark J, Curti BD, Davis E, Kaufman H, Amin A, Alva A, Logan T, Hauke R, Miletello G, Vaishampayan U, Johnson D, White R, Wiernik P, Dutcher J (2017). Long-term disease-free survival (DFS) of metastatic melanoma (mM) and renal cell cancer (mRCC) patients following high-dose interleukin-s (HD IL2). JITC.

[4] Kammula US, White DE, Rosenberg SA (1998). Trends in the safety of high dose bolus interleukin-2 administration in patients with metastatic cancer. Cancer.

[5] Hodi FS (2010). Improved survival with ipilimumab in patients with metastatic melanoma. N Engl J Med.

[6] Postow MA (2015). Nivolumab and ipilimumab versus ipilimumab in untreated melanoma. N Engl J Med.

[7] Topalian SL (2014). Survival, durable tumor remission, and long-term safety in patients with advanced melanoma receiving nivolumab. J Clin Oncol.

[8] Larkin J (2015). Combined Nivolumab and Ipilimumab or monotherapy in untreated melanoma. N Engl J Med.

[9] Robert C (2014). Anti-programmed-death-receptor-1 treatment with pembrolizumab in ipilimumab-refractory advanced melanoma: a randomised dose-comparison cohort of a phase 1 trial. Lancet.

[10] Motzer RJ (2015). Nivolumab versus Everolimus in advanced renal-cell carcinoma. N Engl J Med.

[11] West EE (2013). PD-L1 blockade synergizes with IL-2 therapy in reinvigorating exhausted T cells. J Clin Invest.

[12] Prieto PA (2012). CTLA-4 blockade with ipilimumab: long-term follow-up of 177 patients with metastatic melanoma. Clin Cancer Res.

[13] Weber JS (2016). Sequential administration of nivolumab and ipilimumab with a planned switch in patients with advanced melanoma (CheckMate 064): an open-label, randomised, phase 2 trial. Lancet Oncol.

[14] Clark JI (2018). A multi-center phase II study of high dose interleukin-2 sequenced with vemurafenib in patients with BRAF-V600 mutation positive metastatic melanoma. J Immunother Cancer.

[15] Taniguchi T, Minami Y (1993). The IL-2/IL-2 receptor system: a current overview. Cell.

