# Supplementary material for: Therapy with high-dose Interleukin-2 (HD IL-2) in metastatic melanoma and renal cell carcinoma following PD1 or PDL1 inhibition
Source: J Immunother Cancer. 2019 Feb 18;7:49. doi: 10.1186/s40425-019-0522-3 (PMC6380045; doi:10.1186/s40425-019-0522-3)
Supplement: Supplementary file 1 — Tables S1. Patient Demographics (DOCX 31 kb) [file 40425_2019_522_MOESM1_ESM.docx]

Table 1 Patient Demographics

| Characteristic | mM  (N=40) | mRCC  (N=17) | IL-2 Alone [1]  (N=1122) |
| --- | --- | --- | --- |
|  |  |  |  |
| **Age (yr)** |  |  |  |
| Mean (SD) | 51.6 (10.26) | 54.7 (10.27) | 55.0 (10.13) |
| Median | 53.0 | 55.0 | 56.0 |
| Range | 33-69 | 35-69 | 18-84 |
|  |  |  |  |
| **Gender – no. (%)** |  |  |  |
| Male | 20 (50.0) | 12 (70.6) | 752 (67.0) |
| Female | 17 (42.5) | 4 (23.5) | 369 (32.9) |
|  |  |  |  |
| **Location of metastases – no. (%)** |  |  |  |
| Adrenal Glands | 1 (2.5) | 1 (5.9) | 23 (2.0) |
| Bone | 8 (20.0) | 5 (29.4) | 195 (17.4) |
| Brain | 8 (20.0) | 2 (11.8) | 84 (7.5) |
| Liver | 10 (25.0) | 5 (29.4) | 194 (17.3) |
| Lung(s) | 19 (47.5) | 11 (64.7) | 733 (65.3) |
| Lymph Nodes | 15 (37.5) | 4 (23.5) | 399 (35.6) |
| Pancreas | 1 (2.5) | 0 (0.0) | 14 (1.1) |
| Skin | 7 (17.5) | 1 (5.9) | 77 (6.9) |
| Soft Tissue | 9 (22.5) | 3 (17.6) | 139 (12.4) |
| Stomach | 1 (2.5) | 1 (5.9) | 3 (0.3) |
| Other | 11 (27.5) | 3 (17.6) | 203 (18.1) |
|  |  |  |  |
| **Number of Sites of Metastases – no. (%)** |  |  |  |
| 1 | 1 (2.5) | 3 (17.6) | 418 (37.3) |
| 2 | 6 (15.0) | 3 (17.6) | 357 (31.8) |
| >=3 | 21 (52.5) | 8 (47.1) | 276 (24.6) |
|  |  |  |  |
| **Time between diagnosis of metastatic disease and initiation of IL-2 (months)** |  |  |  |
| Mean (SD) | 22.38 (14.76) | 31.76 (54.47) | 8.24 (16.73) |
| Median | 18.25 | 8.20 | 2.61 |
| Range | 0.8-55.8 | 0.2-155.97 | 0.03-243.34 |
|  |  |  |  |
| **Therapy at Baseline – no. (%)** |  |  |  |
| Drug Therapy | 35 (87.5) | 13 (76.5) | 351 (31.3) |
| Radiation | 18 (45.0) | 4 (23.5) | 223 (19.9) |
| Surgery | 26 (65.0) | 13 (76.5) | 941 (83.9) |
| Ablative Therapy | 1 (2.5) | 0 (0.0) | 4 (0.4) |
| Tumor Infiltrating Lymphocytes | 2 (5.0) | 0 (0.0) | 1 (0.1) |
| Other | 1 (2.5) | 0 (0.0) | 5 (0.4) |
|  |  |  |  |
| **Positive Mutation – no. (%)** |  |  |  |
| BRAF | 4 (10) | 0 (0.0) | 118 (10.5) |
| NRAS | 5 (12.5) | 0 (0.0) | 15 (1.3) |
| cKIT | 1 (2.5) | 0 (0.0) | 7 (0.7) |
|  |  |  |  |
| **Performance Status** |  |  |  |
| 0 | 18 (45.0) | 6 (35.3) | 750 (66.8) |
| 1 | 17 (42.5) | 7 (41.2) | 293 (26.1) |
| 2 | 0 (0.0) | 0 (0.0) | 7 (0.6) |
|  |  |  |  |
