# Supplementary material for: Therapy with high-dose Interleukin-2 (HD IL-2) in metastatic melanoma and renal cell carcinoma following PD1 or PDL1 inhibition
Source: J Immunother Cancer. 2019 Feb 18;7:49. doi: 10.1186/s40425-019-0522-3 (PMC6380045; doi:10.1186/s40425-019-0522-3)
Supplement: Supplementary file 2 — Tables S2. Adverse Events Limiting IL-2 Administration (By Frequency) – supplemental materials (DOCX 32 kb) [file 40425_2019_522_MOESM2_ESM.docx]

Table 2 Adverse Events Limiting IL-2 Administration (By Frequency) – supplemental materials

| Term | mM  (N=40) | mRCC  (N=17) | IL-2 Alone [1]  (N=1122) |
| --- | --- | --- | --- |
|  |  |  |  |
| HYPOTENSION | 12 ( 30.0) | 5 ( 29.4) | 564 ( 50.3) |
| TACHYCARDIA | 5 ( 12.5) | 0 (0.0) | 122 ( 10.9) |
| DIARRHEA | 4 ( 10.0) | 0 (0.0) | 146 ( 13.0) |
| HYPOXIA | 4 ( 10.0) | 0 (0.0) | 48 ( 4.3) |
| THROMBOCYTOPENIA | 4 ( 10.0) | 0 (0.0) | 174 ( 15.5) |
| RIGORS | 3 ( 7.5) | 0 (0.0) | 17 ( 1.5) |
| ACUTE KIDNEY INJURY | 1 ( 2.5) | 1 ( 5.9) | 9 ( 0.8) |
| ACUTE RENAL FAILURE | 0 (0.0) | 2 ( 11.8) | 83 ( 7.4) |
| CAPILLARY LEAK SYNDROME | 2 ( 5.0) | 0 (0.0) | 2 ( 0.2) |
| CONFUSION | 2 ( 5.0) | 0 (0.0) | 148 ( 13.2) |
| DYSPNEA | 1 ( 2.5) | 1 ( 5.9) | 19 ( 1.7) |
| MENTAL FATIGUE | 2 ( 5.0) | 0 (0.0) | 8 ( 0.7) |
| PRURITIS | 2 ( 5.0) | 0 (0.0) | 8 ( 0.7) |
| ACUTE KIDNEY INSUFFIENCY | 1 ( 2.5) | 0 (0.0) | 0 (0.0) |
| ACUTE PULMONARY EDEMA | 1 ( 2.5) | 0 (0.0) | 0 (0.0) |
| ACUTE RENAL INJURY | 1 (0.0) | 1 ( 5.9) | 2 ( 0.2) |
| ARRHYTHMIA | 1 ( 2.5) | 0 (0.0) | 93 ( 8.3) |
| ATRIAL FIBRILLATION | 1 ( 2.5) | 0 (0.0) | 8 ( 0.7) |
| COLITIS | 1 ( 2.5) | 0 (0.0) | 2 ( 0.2) |
| CONSTITUTIONAL AND CNS TOXICITY | 1 ( 2.5) | 0 (0.0) | 0 (0.0) |
| DELIRIUM | 0 (0.0) | 1 ( 5.9) | 5 ( 0.4) |
| DEPRESSION | 0 (0.0) | 1 ( 5.9) | 0 (0.0) |
| DERMATITIS | 1 ( 2.5) | 0 (0.0) | 1 ( 0.1) |
| FEVER | 1 ( 2.5) | 0 (0.0) | 9 ( 0.8) |
| HIVES | 1 ( 2.5) | 0 (0.0) | 1 ( 0.1) |
| HYPERTENSION | 0 (0.0) | 1 ( 5.9) | 0 (0.0) |
| HYPOXIA AND FEVER | 1 ( 2.5) | 0 (0.0) | 0 (0.0) |
| INCREASED PRURITUS | 1 ( 2.5) | 0 (0.0) | 0 (0.0) |
| LOW URINE OUTPUT | 1 ( 2.5) | 0 (0.0) | 19 ( 1.7) |
| METABOLIC ACIDOSIS | 1 ( 2.5) | 0 (0.0) | 0 (0.0) |
| MYOCARDIAL ISCHEMIA | 1 ( 2.5) | 0 (0.0) | 1 ( 0.1) |
| NEUROCORTICAL TOXICITY | 1 ( 2.5) | 0 (0.0) | 0 (0.0) |
| NEUROPATHY IN RIGHT FINGERS | 0 (0.0) | 1 ( 5.9) | 0 (0.0) |
| OLIGURIA | 0 (0.0) | 1 ( 5.9) | 107 ( 9.5) |
| ORAL THRUSH | 1 ( 2.5) | 0 (0.0) | 0 (0.0) |
| PAROXYSMAL ATRIAL FIBRILLATION | 1 ( 2.5) | 0 (0.0) | 1 ( 0.1) |
| RECURRENT RHABDOMYOLYSIS | 1 ( 2.5) | 0 (0.0) | 0 (0.0) |
| RHABDOMYOLYSIS | 1 ( 2.5) | 0 (0.0) | 0 (0.0) |
| RISE IN BILIRUBIN | 1 ( 2.5) | 0 (0.0) | 0 (0.0) |
| SEPTIC SHOCK | 1 ( 2.5) | 0 (0.0) | 0 (0.0) |
| STAPH EPIDERMIS | 1 ( 2.5) | 0 (0.0) | 0 (0.0) |
| VASCULAR LEAK SYNDROME | 0 (0.0) | 1 ( 5.9) | 2 ( 0.2) |

[1] Data only provided where details were presented for either mM or mRCC for comparison.
