# Supplementary material for: Therapy with high-dose Interleukin-2 (HD IL-2) in metastatic melanoma and renal cell carcinoma following PD1 or PDL1 inhibition
Source: J Immunother Cancer. 2019 Feb 18;7:49. doi: 10.1186/s40425-019-0522-3 (PMC6380045; doi:10.1186/s40425-019-0522-3)
Supplement: Supplementary file 3 — Tables S3. Duration of IL-2 Therapy (DOCX 20 kb) [file 40425_2019_522_MOESM3_ESM.docx]

Table 3 Duration of IL-2 Therapy – supplemental material

|  |  |  |
| --- | --- | --- |
|  |  |  |
| Characteristic | mM  (N=40) | mRCC  (N=17) |
|  |  |  |
| Duration of IL-2 Dosing (months) |  |  |
| Mean (SD) | 2.08 (2.09) | 1.79 (1.66) |
| Median | 0.90 | 1.0 |
| Range | 0.1-7.3 | 0.1-6.1 |
|  |  |  |
|  |  |  |
| Total Number of Cycles (one course is two cycles) | | |
| 1 | 8 (20.0) | 2 (11.8) |
| 2 | 11 (27.5) | 6 (35.3) |
| 3 | 3 (7.5) | 0 (0.0) |
| 4 | 9 (22.5) | 5 (29.4) |
| 5 | 0 (0.0) | 0 (0.0) |
| 6 | 4 (10.0) | 1 (5.9) |
| 7 | 1 (2.5) | 1 (5.9) |
|  |  |  |
| Average number of Doses per Cycle |  |  |
| Mean (SD) | 8.07 (2.37) | 7.98 (2.94) |
|  |  |  |
